# The New Texas Medical College

**Published:** 1891-07

**Authors:** 


F. E. DANIEL, M. D., EDITOR AND PUBLISHER.

THIS JOURNAL, by unanimous vote of the members, was made the Official Organ of the Austin District Medical Society.

COLLABORATORS:

*Dr. R. M. Swearingen, Austin.*
*Prof. B. E. Hadra, M. D., Galveston.*
*Prof. Geo. Cupples, M. D., San Antonio.*
*Dr. T. C. Osborn, Cleburne, Texas.*
*Dr E. J. Doering, Chicago.*
*Dr. E. J. Beall, Fort Worth.*
*Dr Odo Betz, Germany.*
*Prof. W. B. Rogers, M. D., Memphis.*
*Dr. J. W. McLaughlin, Austin.*
*Dr. T. J. Tyner, Austin.*
*Prof. J. F. Y. Paine, M. D., Galveston.*
*Dr. R. H. L. Bibb, Mexico.*
*Dr. T. J. Bennett, Austin.*
*Dr. Bat Smith, Wharton.*
*Dr. E. Meierhof, New York.*
*L. H. Luce, M. D., West Tisbury, Mass.*

### THE NEW TEXAS MEDICAL COLLEGE.

The long cherished hope of inaugurating a high grade medical college under the auspices of the State has at last been consummated, and the Medical Department of the University of Texas is fairly launched.

It is cause for rejoicing, indeed. Now the University is complete, and there is not the shadow of an excuse for any of the Texas youth to go beyond the borders of glorious Texas for an education of any kind. Especially was a medical branch needed, inasmuch as statistics showed that as early as 1880 over four hundred young men in Texas studied medicine each year; and they were "paying grist" to other mills. Supposing the number of students to have increased in the same ratio as the population—which in 1880 was 1,500,000, and is now two and three quarter million, the student output should be to 400, as 2¾ is to 1½—about 700; the greater part of which number would, most likely, patronize the home school. In other words—there is ample patronage in Texas to support a flourishing school, even if none were drawn from other sources.

The JOURNAL is pleased to announce that the college building—a handsome modern structure—heretofore described in these pages—is about completed; it will be ready for occupancy Oct. 4th, the opening day of the first session. There are to be eight chairs; i. e. in addition to the regular seven branches there will be a chair of Pathology, Histology and Bacteriology. Only two chairs have as yet been filled, to wit: that of Obstetrics and Gynecology, for which Prof. J. F. Y. Paine has been selected,—than whom a more able, courteous and popular man could not be found,—the very man to shed lustre upon the institution; and Prof. Hamilton A. West, the popular Secretary of the Texas State Medical Association, and who has so long held the corresponding chair in the Galveston Medical College, has been elected to the chair of Theory and Practice of Medicine. This is certainly a high testimonial to Dr. West's merit and ability, one upon which he is to be sincerely congratulated.

The Board of Regents will meet in Galveston on the last Monday in August to fill the remaining chairs; and for the vacancies there are numerous applications—one, the JOURNAL is informed, from Belfast, Ireland, and one from Edinburgh, Scotland. For the chair of Chemistry, amongst others, Dr. Seth M. Morris, of Austin, is an applicant. Dr. Morris is one of the most thorough chemists in Texas, he having been chosen by Prof. Everhart, the chemist of the University, to assist him the last year of his student term—he, Dr. Morris, being a graduate of the University, and a native Texan; and when in his senior year, and at the age of twenty-one, he had the endorsement of the Faculty for appointment as chemist to the Agricultural and Mechanical College at Bryan. Dr. Morris is strongly endorsed by Dr. Everhart, the Chemist, and Dr. McFarland, Professor of Physics. Dr. Wysong, who has held the Chair of Chemistry in the Galveston Medical College, is also an applicant, and brings strong endorsement. We learn that Dr. Paul Paguin, of Missouri, is an applicant for the new chair, and that Dr. Geo. Dock, the late Pathologist of the Galveston school, has accepted a position at Ann Arbor, Mich.

The salaries to be paid the professors in the new medical college range from $2000 to $3000. The curriculum will be graded, three courses of lectures, of seven months each, will be required to complete it. As to the requirements for matriculation the JOURNAL is not yet informed, but it assures its readers that the college will be in every respect high grade, and fully up to the requirements of a thorough modern medical education. This is expected,—demanded,—and the profession and the people will be satisfied with nothing less. It will be the aim to make a diploma bearing the "Lone Star" a coveted prize and a passport to the highest and most cultured medical circles in the world. Attention is called to the announcement in the advertising pages.

